# Revisiting Cytomegalovirus Serology in Allogeneic Hematopoietic Cell Transplant Recipients

**DOI:** 10.1093/cid/ciad550

**Published:** 2023-09-15

**Authors:** Vera Portillo, Stavroula Masouridi-Levrat, Léna Royston, Sabine Yerly, Manuel Schibler, Maria Mappoura, Sarah Morin, Federica Giannotti, Anne-Claire Mamez, Christian van Delden, Yves Chalandon, Dionysios Neofytos

**Affiliations:** Division of Infectious Diseases, University Hospital of Geneva, Geneva, Switzerland; Division of Hematology, Bone Marrow Transplant Unit, University Hospital of Geneva and Faculty of Medicine, University of Geneva, Geneva, Switzerland; Division of Infectious Diseases, University Hospital of Geneva, Geneva, Switzerland; Laboratory of Virology, Laboratory Medicine Division, University Hospital of Geneva, Geneva, Switzerland; Division of Infectious Diseases, University Hospital of Geneva, Geneva, Switzerland; Laboratory of Virology, Laboratory Medicine Division, University Hospital of Geneva, Geneva, Switzerland; Division of Hematology, Bone Marrow Transplant Unit, University Hospital of Geneva and Faculty of Medicine, University of Geneva, Geneva, Switzerland; Division of Hematology, Bone Marrow Transplant Unit, University Hospital of Geneva and Faculty of Medicine, University of Geneva, Geneva, Switzerland; Division of Hematology, Bone Marrow Transplant Unit, University Hospital of Geneva and Faculty of Medicine, University of Geneva, Geneva, Switzerland; Division of Hematology, Bone Marrow Transplant Unit, University Hospital of Geneva and Faculty of Medicine, University of Geneva, Geneva, Switzerland; Division of Infectious Diseases, University Hospital of Geneva, Geneva, Switzerland; Division of Hematology, Bone Marrow Transplant Unit, University Hospital of Geneva and Faculty of Medicine, University of Geneva, Geneva, Switzerland; Division of Infectious Diseases, University Hospital of Geneva, Geneva, Switzerland

**Keywords:** CMV, CMV serology, false-positive, letermovir‌, allogeneic hematopoietic cell transplantation

## Abstract

**Background:**

Allogeneic hematopoietic cell transplant recipients (allo-HCTRs) with positive cytomegalovirus (CMV) serology may have false-positive results due to blood product transfusion–associated passive immunity.

**Methods:**

This single-center cohort study included allo-HCTRs with negative baseline (at malignancy diagnosis) CMV serology and indeterminate/low-positive (CMV IgG titer, ≥0.6–<50 U/mL) pretransplant CMV serology with negative pretransplant plasma CMV DNAemia. The CMV status of those patients was reclassified from R+ to R− (CMVR− reclassification group). We compared those patients to allo-HCTRs with negative (CMV IgG titer <0.6 U/mL) pretransplant CMV IgG (CMVR− group). We describe the number and type of patients whose pretransplant CMV status was reclassified from indeterminate/positive to negative. We reviewed all plasma CMV DNAemia tests performed during the first 6 months posttransplant in both groups to assess the safety of this approach.

**Results:**

Among 246 (84.5%) of 291 transplanted patients identified as CMVR+ pretransplant, 60 (24.4%) were reclassified from CMV serology indeterminate (N:10)/low-positive (N:50) to R−. Only 1 of 60 patients (1.67%) in the CMVR− reclassification group versus 3 of 44 (6.8%; *P* = .30) in the CMVR− group developed CMV DNAemia during the follow-up period. There were no significant differences in the number of CMV DNAemia tests performed, CMV DNAemia range, and time posttransplant between the 2 groups.

**Conclusions:**

One of 4 allo-HCT CMVR+ may be falsely flagged as R+, with significant impact on donor selection and prophylaxis administration. A 2-step approach including CMV serology testing at hematologic malignancy diagnosis in allo-HCT candidates and careful review of pretransplant CMV IgG titers may help correctly classify CMV serology status.

## Teaser

Indeterminate/low-positive CMVIgGtiters pretransplant may signify transfusion-related false-positive CMV status in 1 of 4 allo-HCT recipients, with an impact on donor selection and prophylaxis. We propose anapproach including CMV serology at malignancy diagnosis and pretransplant CMV IgG titer review.

## Graphical Abstract

**Graphical Abstract:**
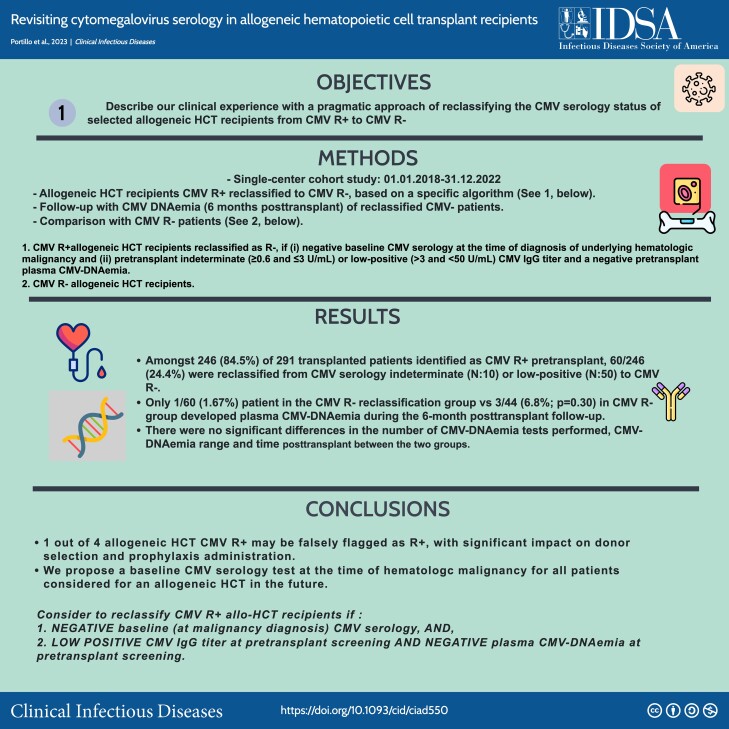
This graphical abstract is also available at Tidbit: https://tidbitapp.io/tidbits/revisiting-cytomegalovirus-serology-in-allogeneic-hematopoietic-cell-transplant-recipients-e66f73fb-8238-44cb-a4b4-b9859f8c7682

Cytomegalovirus (CMV) serology status of allogeneic hematopoietic cell transplant recipients (allo-HCTRs) is an important variable with potential implications on the selection of a suitable donor but also on administration of primary CMV prophylaxis with letermovir in CMV-positive recipients (CMV R+) [1–4]. Recent data suggest that almost 1 in 4 allogeneic HCTRs who tested positive for CMV serology may have false-positive results [5, 6]. This may, in part, be explained by passive immunity from previous transfusions of blood products [5, 7]. In a recently published Good Practice Paper by the British Society for Hematology, it is suggested that all potential HCTRs should have CMV serology testing at the time of diagnosis of their underlying hematologic malignancy and prior to blood transfusions to avoid clinically false-positive CMV serology results [5].

We have observed that several allo-HCTRs with negative CMV serology at the time of diagnosis of their hematology malignancy diagnosis had a positive CMV serology upon their pretransplant evaluation, often with low CMV immunoglobulin G (IgG) titers, which could have been passively transferred during transfusion of blood products between underlying hematologic malignancy diagnosis and transplantation. Using selective criteria, we reclassified some of those patients as CMV R-negative (R−) for their allo-HCT. Here, we describe our clinical experience with a pragmatic approach of reclassifying the CMV serology status of carefully selected allo-HCTRs from CMV R+ to CMV R−.

## METHODS

### Study Design

In a single-center cohort of consecutive adult (aged ≥18 years) allo-HCTRs between 1 January 2018 and 31 December 2022, all patients with a negative CMV serology at the time of their underlying hematologic malignancy diagnosis and an indeterminate or positive CMV serology at the time of their pretransplant consultation were reviewed. Among them, patients who had a recent (within 6 months prior) negative baseline CMV serology at the time of diagnosis of the underlying hematologic malignancy, a pretransplant indeterminate (between ≥0.6 and ≤3 U/mL) or low-positive (arbitrarily defined between >3 and <50 U/mL) CMV IgG titer, and a negative pretransplant plasma CMV DNAemia were reclassified as CMV R− and included in this study (CMV R− reclassification group). We compared those patients to allo-HCTRs who had a negative (<0.6 U/mL) pretransplant CMV IgG serology (CMV R− group). The primary objective was to describe the number and characteristics of patients whose pretransplant CMV serology status was reclassified from indeterminate or positive to negative. In addition and in order to assess the safety of this approach, we reviewed all plasma CMV DNAemia tests performed during the first 6 months posttransplant in both patient groups. The study was approved by the local ethics committee.

### Data Collection

Data on the following variables were collected: demographics, underlying disease, and HCT-associated variables including conditioning regimen, type of donor, and HCT source. The following CMV-associated variables were also collected: D/R (donor/recipient) CMV pretransplant serology, R CMV serology at malignancy diagnosis when available, and plasma CMV DNAemia during the first 6 months after HCT including number of tests performed, number of positive tests per patient, CMV DNAemia viral load, and time from transplant to first positive CMV DNAemia.

### CMV Serology and DNAemia Testing Institutional Standard Operating Procedures and Definitions

All allogeneic HCT candidates at our institution have a transplant infectious disease consultation within approximately 4 weeks, as previously described (V Portillo, et al, manuscript submitted, under review). Pretransplant CMV serology and plasma CMV DNAemia are systematically performed at the time of this pretransplant consultation. Furthermore, CMV serology status at the time of diagnosis of underlying hematologic malignancy, defined here as “baseline” CMV serology, was reviewed when available. CMV serology testing was performed using Elecsys CMV IgG (Roche Diagnostics, Rotkreuz, Switzerland), with a negativity cutoff <0.6 U/mL, indeterminate results between ≥0.6 and ≤3 U/mL, and positive results >3 U/mL (range of quantification, 0–500 U/mL). CMV DNAemia was measured in plasma using a quantitative polymerase chain reaction (qPCR) assay with the COBAS CMV for Cobas 6800 test (Roche Diagnostics, Indianapolis, IN) since 16 May 2018. The limit of detection (LOD) and the limit of quantification (LOQ) of this assay are 22 IU/mL and 25 IU/mL, respectively [8, 9]. Previously, CMV qPCR was performed with the COBAS AmpliPrep/COBAS TaqMan CMV test (Roche Diagnostics, Indianapolis, IN), which displayed a LOD and LOQ of 56 IU/mL and 137 IU/mL, respectively. Plasma CMV DNAemia was measured once weekly for the first 3 months and bimonthly between 4 and 6 months posttransplant in all D+R− and R+ allo-HCTRs. In CMV D−R− allo-HCTRs, CMV DNAemia was measured as for D+R−/R+ patients until 28 May 2019, once monthly for the first 6 months between 28 May 2019 and 1 March 2022, and based on clinical suspicion after 1 March 2022. Letermovir primary CMV prophylaxis was administered to CMV serology D−/R+ from 1 May 2019 to 31 December 2020 and starting 1 January 2021 to all CMV R+ adult allo-HCTRs from post-HCT day 1 to day 100. Primary prophylaxis with letermovir was also administered to all adult CMV R+ with early (during the first 6 months post-HCT) grade ≥2 acute graft-vs-host disease that required corticosteroid treatment at a dose of ≥1 mg/kg/d and until tapering to <10 mg/d of prednisone equivalent since 01 May 2019 [9]. None of the recipients with reclassification of their CMV serology from indeterminate/positive to negative received prophylaxis with letermovir. Positive plasma CMV DNAemia was defined as either a detectable and/or quantifiable test result.

### Statistical Analyses

Study data were collected and managed using REDCap (Research Electronic Data Capture) tools hosted at Geneva University Hospital [10, 11]. Descriptive statistics were used to characterize the study sample. The median and interquartile range (IQR) were calculated to describe continuous variables and frequencies and percentages for categorical variables. Categorical and continuous variables were compared using the Fisher exact and a 2-tailed Student *t* test, as appropriate. Statistical analyses were performed using Stata Statistical Software, release 16 (StataCorp, College Station, TX), and figures were generated using GraphPad Prism 8.0 (GraphPad Software, Inc., San Diego, CA).

## RESULTS

**Figure 1.**
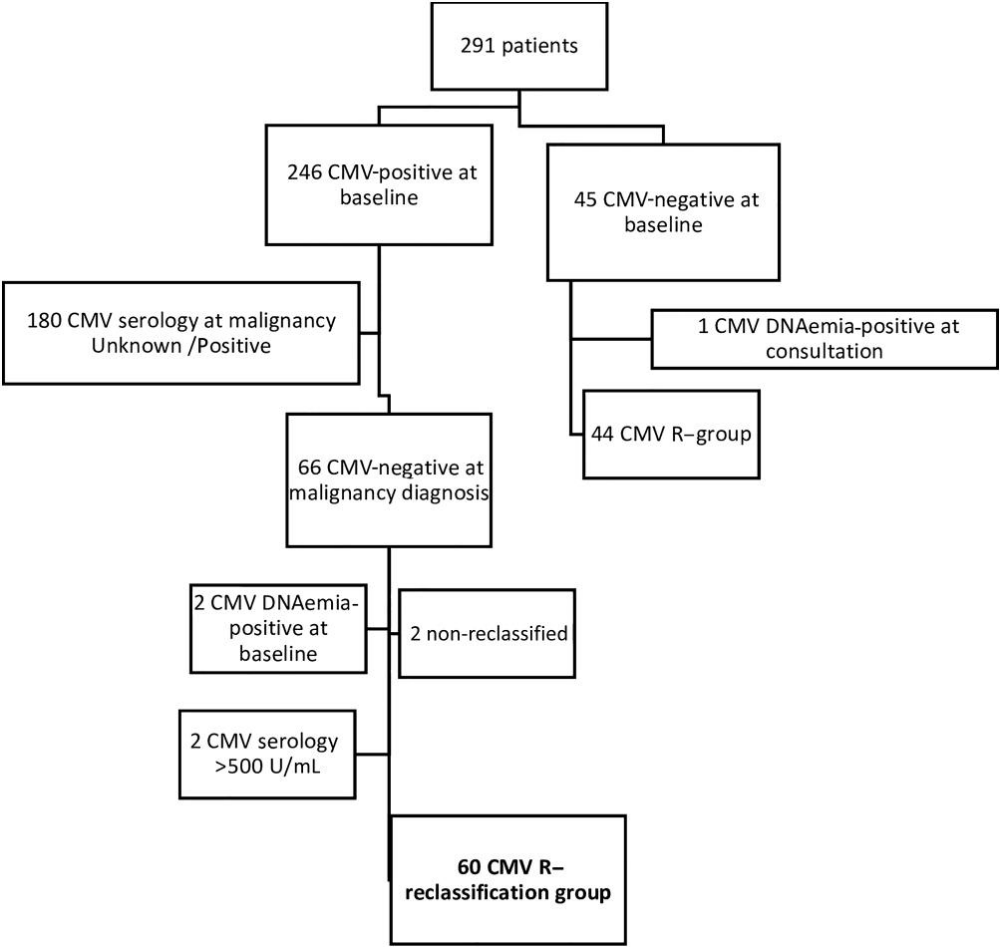
Study flowchart. Abbreviation: CMV, cytomegalovirus.

There were 291 patients transplanted with available pretransplant CMV serology during the study period, with 246 of 291 (84.5%) patients identified as CMV R+ at the time of their pretransplant evaluation. Among the latter, 60 of 246 (24.4%) patients had a negative baseline CMV serology, indeterminate (N, 10) or low-positive (N, 50) CMV IgG titer, and negative plasma CMV DNAemia at the time of the pretransplant consultation and were reclassified from CMV R+ to R− and included in this study accordingly (Figure 1).

### CMV R− Reclassification Group

The patient baseline characteristics are detailed in Table 1. Fifty (83.3%) patients had a pretransplant CMV IgG titer between >3 and <50 U/mL considered as low-positive, and 10 (16.7%) patients had an indeterminate CMV IgG titer between 0.6 and 3 U/mL. There were no significant differences between patients with indeterminate and low-positive CMV serology in terms of demographics and HCT-associated variables. Lymphoma was more prevalent in patients with indeterminate CMV serology (3 of 10, 30% vs 2 of 50, 4%; *P* = .03). The median pretransplant CMV serology titer was 6.8 U/mL (IQR, 3.5–44.1; Supplementary Figure 1*A*): higher in patients with low-positive (7.9 U/mL; IQR, 5.7–14.5) compared with patients with indeterminate (1.8 U/mL; IQR, 1.1–2.3; *P* > .01) CMV serology. Donors had a negative CMV serology in 46 of 60 (76.7%) cases.

**Table 1.** Patient Characteristics for 60 Allogeneic Hematopoietic Cell Transplant Recipients Whose Cytomegalovirus (CMV) Serology Was Reclassified From Positive to Negative and 44 Patients With True Negative CMV Serology

	CMV R− Reclassification Group, N = 60 (%)					
Characteristic	Low-Titer Positive CMV Serology, N = 50 (%)	Indeterminate CMV Serology, N = 10 (%)	*P* Value^b^	All Patients N = 60 (%)	CMV R− Group N = 44 (%)	*P* Value^a^
Demographics						
** **Age, median (IQR), y	55 (44–66)	57.5 (53–63)	.87	55 (46–65)	62.5 (53.5–69.5)	.07
** **Gender, male	28 (56)	6 (60)	1	34 (56.7)	31 (70.4)	.22
Underlying disease			.03			<.01
Acute myelogenous leukemia/ Myelodysplastic syndrome	41 (82)	7 (70)		48 (80)	26 (59.1)	
** **Lymphoma	2 (4)	3 (30)		5 (8.3)	10 (22.7)	
** **Acute lymphoblastic leukemia	5 (10)	0		5 (8.3)	0	
** **Chronic leukemia^c^	0	0		0	6 (13.6)	
** **Multiple myeloma	2 (4)	0		2 (3.3)	2 (4.5)	
HCT-associated variables						
** **Conditioning regimen, reduced intensity	35 (70)	7 (70)	1	42 (70)	37 (84.1)	.16
Donor			.36			.78
** **Matched unrelated donor	24 (48)	5 (50)		29(48.3)	21 (47.7)	
** **Matched related donor	14 (28)	1 (10)		15 (25)	8 (18.2)	
** **Haploidentical donor	9 (18)	4 (40)		13 (21.7)	12 (27.3)	
** **Mismatched unrelated donor	3 (6)	0		3 (5)	3 (6.8)	
HCT source, peripheral blood stem cells	50 (100)	9 (90)	.16	59 (98.3)	42 (95.4)	.57
Pretransplant CMV immunoglobulin G, median (IQR), U/mL	7.9 (5.7–14.5)	1.8 (1.1–2.3)	<.01	6.8 (3.5–44.1)	N/A	N/A
CMV donor serology			.68			1
** **Negative	39 (78)	7 (70)		46 (76.7)	33 (75)	
** **Positive	11 (22)	3 (30)		14 (23.3)	11 (25)	

Abbreviations: CMV, cytomegalovirus; HCT, hematopoietic cell transplant; IQR, interquartile range; NA, not applicable; R, recipient.

^a^ *P* value compares 60 allogeneic HCT recipients whose pretransplant CMV serology was reclassified from positive to negative with 44 patients with negative CMV serology.

^b^ *P* value compares allogeneic HCT recipients with indeterminate (CMV immunoglobulin G ≥1 to ≤3 U/mL) versus low-positive (>3 to <50 U/mL) CMV serology whose pretransplant CMV serology was reclassified from positive to negative.

^c^There were 2 cases of chronic myelogenous and 4 cases of lymphoid leukemia.

Only 1 patient (1 of 60, 1.7%) developed a positive plasma CMV DNAemia during 6-month follow-up posttransplant in the CMV R− reclassification group (Table 2). This patient was aged <30 years with a pretransplant CMV serology IgG titer of 13.8 U/mL and a CMV D+. The patient engrafted on posttransplant day 15 and was discharged home on post-HCT day 30. The first positive CMV DNAemia was observed 37 days posttransplant, with a maximum value of 66 100 IU/mL and 11 positive PCR tests overall (Supplementary Figure 1*B*). The patient was treated with valganciclovir and had resolution of CMV DNAemia within 44 days after preemptive CMV treatment initiation without developing CMV-associated end-organ disease. Rather than a reactivation, a donor-associated or primary CMV infection was clinically suspected in this case.

**Table 2.** Plasma Cytomegalovirus (CMV) DNAemia Results During the First 6 Months Posttransplant for 60 Allogeneic Hematopoietic Cell Transplant Recipients With Positive CMV Immunoglobulin G Serology Titer Between ≥0.6 and <50 U/mL and 44 Patients With Negative Pretransplant CMV Serology

	CMV R− Reclassification Group, N = 60 (%)					
Plasma CMV DNAemia Results^a^	Low-Titer Positive CMV Serology, N = 50 (%)	Indeterminate CMV Serology, N = 10 (%)	*P* Value^c^	All R− Reclassification Group, N = 60 (%)	CMV R− Group, N = 44 (%)	*P* Value^b^
No. of tests performed per patient, median (IQR)	15.5 (11–20)	18 (12–19)	.76	16.5 (11.5–19.5)	15 (12–21)	.69
No. of patients with any positive plasma CMV DNAemia (nonquantifiable included)^d^	1 (2)	0	N/A	1(1.7)	3 (6.8)	N/A
No. of patients with positive detectable plasma CMV DNAemia per patient (only quantifiable)	1 (2)	0	N/A	1 (1.7)	2 (4.5)	N/A
Plasma CMV DNAmia range, median (IQR), U/mL	1500 (140–4200)	0	N/A	1500 (140–4200)	874 (182–1895)	.26
No. of positive CMV DNAemia per patient, median (IQR), U/mL	11 (11–11)	…		11 (11–11)	7 (2–12)	.56
Days posttransplant for first positive plasma CMV DNAmia, median (IQR)	37 (37–37)	N/A	N/A	37 (37–37)	35 (25–41)	N/A

Abbreviations: CMV, cytomegalovirus; IQR, interquartile range; N/A, not applicable; R, recipient.

^a^Plasma CMV DNAmia results presented here refer to tests performed during the first 6 months posttransplant with the COBAS CMV for Cobas 6800 test (Roche Diagnostics, Indianapolis, IN) with ≥22 and ≥25 IU/mL for level of detection (LOD) and level of quantification (LOQ), respectively [7,8], since 16 May 2018. Of note, 7 patients received transplants between 1 January 2018 and 16 May 2018 when plasma CMV DNAemia was measured with the COBAS AmpliPrep/COBAS TaqMan CMV test (Roche Diagnostics, Indianapolis, IN) with an LOD and LOQ of 56 and 137 IU/mL, respectively.

^b^ *P* value compares 60 allogeneic hematopoietic cell transplant (HCT) recipients whose pretransplant CMV serology was reclassified from positive to negative with 44 patients with negative CMV serology.

^c^ *P* value compares allogeneic HCT recipients with indeterminate (CMV immunoglobulin G ≥0.6 to ≤3 U/mL) versus low-positive (>3 to <50 U/mL) CMV serology whose pretransplant CMV serology was reclassified from positive to negative.

^d^Results are presented per patient; each patient is counted once, regardless if plasma CMV DNAemia was positive more than once per patient.

### CMV R− Group

There were 45 patients who were CMV R− at pretransplant screening, 1 of whom with positive plasma CMV DNAemia. Hence, we included 44 patients with negative CMV serology status and negative plasma CMV DNAemia. Baseline CMV serology status was known for 28 of 44 (63.6%) patients and was negative in 27 of 28 (96.4%) of them. The 1 patient with positive baseline CMV serology had a negative pretransplant CMV serology test and was considered CMV R−, did not receive letermovir prophylaxis, and did not develop CMV reactivation posttransplant. Whether this was a false-positive at baseline or false-negative pretransplant test could not be confirmed retrospectively in this case. Patient characteristics in this group were similar to those of patients in the CMV R− reclassification group in terms of demographics and HCT-associated variables (Table 1). However, patients in the CMV R− group were less likely (26 of 44, 59.1%) to have an underlying diagnosis of acute myeloid leukemia/myelodysplastic syndrome compared with the CMV R− reclassification group (49 of 61, 80.3%; *P* < .01). Donors were CMV D− in 33 of 44 (75%) allo-HCTRs in this group.

In the CMV R− group, there were a median of 15 (IQR, 11–19) plasma CMV DNAemia tests performed per patient, similar to 16.5 (IQR, 11.5–19.5) tests performed per patient in the CMV R− reclassification group (*P* = .69; Table 2). Three patients had positive plasma CMV DNAemia during the first 6-month posttransplant follow-up period: 2 with at least 1 CMV quantifiable CMV DNAemia and 1 with only 2 plasma detectable and no quantifiable CMV DNAemia. Two of them had unknown baseline CMV serology; the third had a negative baseline CMV serology, and the donor was positive in 2 patients. There were 11 positive plasma CMV DNAemia tests per patient in the CMV R− reclassification group and a median of 7 [2, 12] in the CMV R− group. Plasma CMV DNAemia was positive at a median of 35 (IQR, 25–41) days posttransplant with a maximal value of 874 U/mL and 6590 U/mL in the 2 patients with quantifiable CMV DNAemia.

## DISCUSSION

Our data suggest that 1 of 4 allogeneic HCT CMV R+ may be falsely flagged as R+, with potential significant impact on CMV donor selection, prophylaxis administration, and clinical outcomes. Consistent with reports by the British Society for Hematology, we observed a number of allogeneic HCT candidates to have low (either indeterminate or low-positive) CMV IgG titers in the setting of negative prior CMV serology, considering the former as a potential effect of passive immunity from previous transfusions of blood products [5, 7]. Using specific criteria, as described above, some of those patients were reclassified from R+ to R− and considered as such throughout their transplant. With an intensive follow-up, only 1 of those patients developed CMV reactivation during the first 6 months posttransplant. This was an infection that was diagnosed about a month posttransplant after the patient's discharge. Although it could be associated with the patient's CMV-positive donor, a primary CMV infection was suspected, as suggested by the out-of-range high plasma CMV DNAemia >1 month posttransplant and after hospital discharge timing. The rest of the patients with reclassified negative CMV serology remained with negative plasma CMV DNAemia for the first 6 months posttransplant without any evidence of CMV reactivation or infection. This was similar between patients with indeterminate and low-positive CMV serology.

To further validate our approach, we compared those patients with all CMV R− in this cohort, considered as a “gold standard” group. Both groups had a similar number of plasma CMV DNAemia tests performed during the study follow-up period. CMV DNAemia rates between the CMV R− reclassification and CMV R− groups were similar, suggesting that patients in the former group present a behavior analogous to the CMV R− “true-negative” patients. CMV DNAemia in this group could be attributed to either a CMV D+ status (at least in 2 of 3 cases) or potentially false-negative pretransplant CMV serology results. Notably, more patients in the CMV R− group were likely to have an underlying diagnosis of lymphoma, admittedly less frequently transfused than patients with myeloid hematologic malignancies. Furthermore, patients with lymphoma and other hematologic malignancies could have impaired humoral immune responses as a result of their malignancy and associated administered treatments [12]. It is also possible that some of those CMV R− patients were “false-negative” due to chemotherapy-induced antibody loss. This observation further emphasizes the importance of baseline CMV serology at the time of hematologic malignancy diagnosis. Similar observations can be made in the interpretation of other serology tests in the same context. For instance, we have described in the same cohort of patients the reclassification of positive *Toxoplasma gondii*, hepatitis virus A and/or B serology in 22 of 292 (7.5%) and 38 of 292 (13%), respectively (V Portillo, et al, manuscript submitted, under review). The above suggests that careful review of serology titers pretransplant and comparison, if and when feasible, with values at the time of hematologic malignancy diagnosis may allow for correct classification of patients' serology status.

The clinical implications of our findings in the management of allo-HCTRs are many. First, CMV D+ has been historically prioritized over CMV D− for CMV R+ to decrease the risk of CMV infection posttransplant. Although this approach may no longer be applied in many transplant centers where letermovir prophylaxis is routinely used, it may still have an impact on donor selection in countries and settings where anti-CMV prophylaxis is either not available or not routinely applied. In our cohort, almost 2 of 3 donors were CMV D− in the CMV R− reclassification group, which is similar to donors in the CMV R− group. Furthermore, in centers with routine administration of primary CMV prophylaxis with letermovir in allo-HCTRs, reclassifying a CMV R+ to R− could lead to potential lower drug use and significant cost reduction. We previously reported an estimated mean cost for letermovir administration of $38 500 per patient [8]. Reclassification of 60 patients as CMV R− allowed us to spare more than $2 million only in primary anti-CMV prophylactic treatment. Furthermore, considering the less frequent monitoring of plasma CMV DNAemia follow-up for CMV R−/D−, additional potential savings in costs and virology laboratory resource utilization could be anticipated.

Our study had many limitations. Baseline CMV serology was not available for all patients, and the IgG titer cutoff of 50 U/mL for low-positive CMV serology was arbitrarily chosen based on our observations and clinical experience. Notably, the CMV IgG titers used in this study may not necessarily be applicable in all centers where various serology tests are used. Data on the administration of blood transfusions (units and timing pretransplant) between the diagnosis of underlying hematologic malignancy and transplantation were not available. Finally, CMV serology avidity testing was not performed or requested in any of the patients included in this cohort; hence, additional recommendations with regard to the use of this test are not feasible.

**Figure 2.**
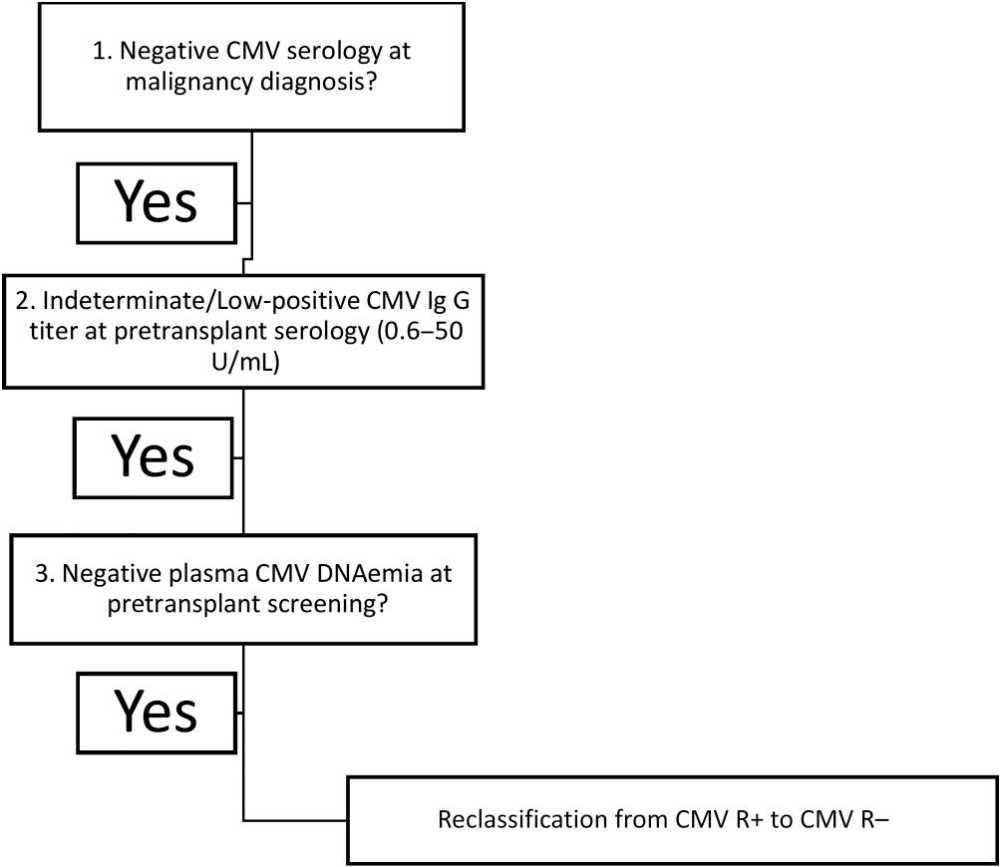
Proposed algorithm to safely reclassify allogeneic hematopoietic cell transplant recipients from CMV-positive to CMV-negative, in case of baseline (at the time of diagnosis of hematologic malignancy) negative CMV serology, indeterminate or low-positive pretransplant CMV IgG titers, and negative pretransplant plasma CMV DNAemia. Abbreviations: CMV, cytomegalovirus; IgG, immunoglobulin G.

In conclusion, our findings could be considered as a proof of concept to validate a 2-step approach with routine baseline CMV serology for all allogeneic HCT candidates at the time of hematologic malignancy diagnosis and for those patients with negative baseline CMV serology, a careful review of pretransplant CMV serology IgG titers and DNAemia for identification of potential false-positive results (Figure 2). Our observations are aligned with the British Society for Hematology suggestions, indicating that all potential HCT recipients should have CMV serology testing at the time of diagnosis of their underlying hematologic malignancy, which we believe should be an integral tool in the interpretation of CMV serology in HCT candidates. Considering the increasing number of HCTs successfully performed all over the world, larger prospective studies and algorithm propositions, such as ours, are necessary to optimize clinical outcomes and resource utilization. In addition, recent data suggest that the higher the CMV serology titer of an allogeneic HCT CMV R+, the higher the probability of posttransplant CMV reactivation [13, 14]. Clearly, the importance and clinical impact of CMV serology in allo-HCTRs requires a second fresh look.

## Supplementary Data

Supplementary materials are available at *Clinical Infectious Diseases* online. Consisting of data provided by the authors to benefit the reader, the posted materials are not copyedited and are the sole responsibility of the authors, so questions or comments should be addressed to the corresponding author.

## Supplementary Material

ciad550_Supplementary_Data

## Notes

***Author Contributions.*** V. P., S. M.-L., and D. N. conceived the idea for the study and the principal study design. All other authors contributed data. V. P. and D. N. analyzed the data and wrote the manuscript. All co-authors reviewed the manuscript, provided suggestions, and gave their final approval of the manuscript.

***Acknowledgments.*** The authors thank all patients and staff involved in the care of patients at the Bone Marrow Unit at Geneva University Hospitals.

***Data availability statement.*** The datasets analyzed for this study are available from the corresponding author upon request.
