# Supplementary material for: Revisiting Cytomegalovirus Serology in Allogeneic Hematopoietic Cell Transplant Recipients
Source: Clin Infect Dis. 2023 Sep 15;78(2):423–9. doi: 10.1093/cid/ciad550 (PMC10874258; doi:10.1093/cid/ciad550)
Supplement: ciad550_Supplementary_Data [file ciad550_supplementary_data.docx]

**
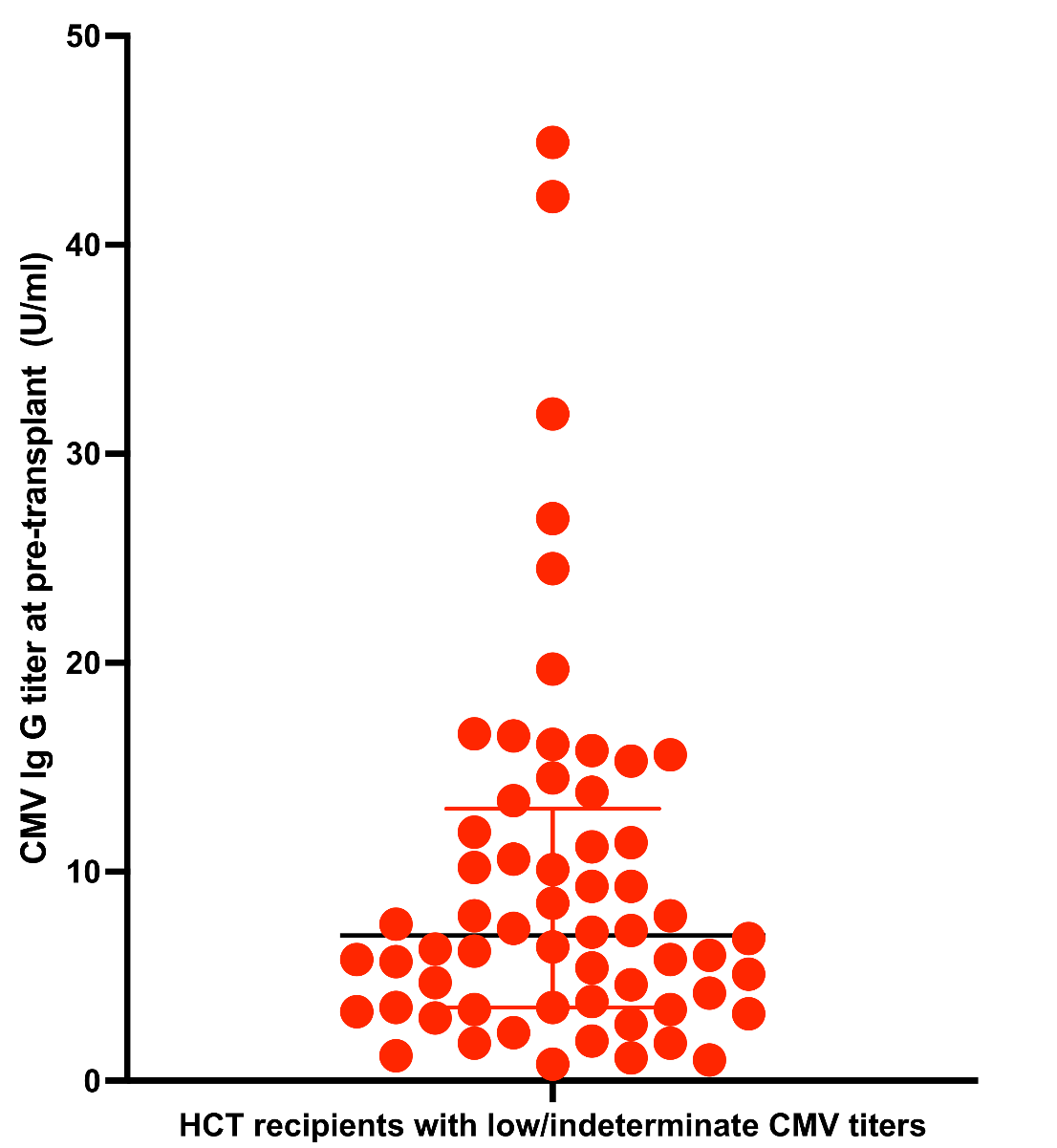
**

**Supplement Figure 1a.** Pretransplant CMV IgG serology titers in 60 allogeneic hematopoietic cell transplant recipients with indeterminate (≥0.6 to ≤3 U/mL) and low-positive (>3 to <50 U/mL) pretransplant CMV IgG serology titer who were reclassified as CMV R-. Each dot represents one patient. CMV IgG was performed with Enzyme immunoassay (EIA), Elecsys, Roche, Switzerland.

**Supplement Figure 1b.** Number of positive plasma CMV DNAemia tests in patients who had a positive DNAemia during the follow-up, in the CMV R- reclassification group (1 patient) and the CMV R- group (3 patients). Each dot represents one patient, while the y-axis represents the number of positive (detectable and/or quantifiable) plasma CMV DNAemia results per patient. CMV DNAemia was performed with the COBAS Ampli/Prepe/COBAS TaqMan CMV test (Roche Diagnostics, Indianapolis, IN, USA) until May 2018. After May 2018 COBAS CMV for Cobas 6800 test (Roche Diagnostics, Indianapolis, IN, USA).
